# Persistence and decay of human antibody responses to the receptor binding domain of SARS-CoV-2 spike protein in COVID-19 patients

**DOI:** 10.1126/sciimmunol.abe0367

**Published:** 2020-10-08

**Authors:** Anita S. Iyer, Forrest K. Jones, Ariana Nodoushani, Meagan Kelly, Margaret Becker, Damien Slater, Rachel Mills, Erica Teng, Mohammad Kamruzzaman, Wilfredo F. Garcia-Beltran, Michael Astudillo, Diane Yang, Tyler E. Miller, Elizabeth Oliver, Stephanie Fischinger, Caroline Atyeo, A. John Iafrate, Stephen B. Calderwood, Stephen A. Lauer, Jingyou Yu, Zhenfeng Li, Jared Feldman, Blake M. Hauser, Timothy M. Caradonna, John A. Branda, Sarah E. Turbett, Regina C. LaRocque, Guillaume Mellon, Dan H. Barouch, Aaron G. Schmidt, Andrew S. Azman, Galit Alter, Edward T Ryan, Jason B. Harris, Richelle C. Charles

**Affiliations:** a Division of Infectious Diseases, Massachusetts General Hospital, Boston, MA, USA.; b Department of Medicine, Harvard Medical School, Boston, MA, USA.; c Department of Epidemiology, Johns Hopkins Bloomberg School of Public Health, Baltimore, MD, USA.; d Department of Pathology, Massachusetts General Hospital, Boston, MA, USA.; e Ragon Institute of MGH, MIT, and Harvard, Cambridge, MA, USA.; f Department of Microbiology, Harvard Medical School, Boston, MA, USA.; g Center for Virology and Vaccine Research, Beth Israel Deaconess Medical Center, Harvard Medical School, Boston, MA, USA.; h Department of Immunology and Infectious Diseases, Harvard T.H. Chan School of Public Health, Boston, MA, USA.; i Department of Pediatrics, Harvard Medical School, Boston, MA, USA.

## Abstract

We measured plasma and/or serum antibody responses to the receptor-binding domain (RBD) of the spike (S) protein of SARS-CoV-2 in 343 North American patients infected with SARS-CoV-2 (of which 93% required hospitalization) up to 122 days after symptom onset and compared them to responses in 1548 individuals whose blood samples were obtained prior to the pandemic. After setting seropositivity thresholds for perfect specificity (100%), we estimated sensitivities of 95% for IgG, 90% for IgA, and 81% for IgM for detecting infected individuals between 15 and 28 days after symptom onset. While the median time to seroconversion was nearly 12 days across all three isotypes tested, IgA and IgM antibodies against RBD were short-lived with median times to seroreversion of 71 and 49 days after symptom onset. In contrast, anti-RBD IgG responses decayed slowly through 90 days with only 3 seropositive individuals seroreverting within this time period. IgG antibodies to SARS-CoV-2 RBD were strongly correlated with anti-S neutralizing antibody titers, which demonstrated little to no decrease over 75 days since symptom onset. We observed no cross-reactivity of the SARS-CoV-2 RBD-targeted antibodies with other widely circulating coronaviruses (HKU1, 229 E, OC43, NL63). These data suggest that RBD-targeted antibodies are excellent markers of previous and recent infection, that differential isotype measurements can help distinguish between recent and older infections, and that IgG responses persist over the first few months after infection and are highly correlated with neutralizing antibodies.

## Teaser

IgM and IgA responses to SARS-CoV-2 RBD in severe COVID patients decay rapidly, while IgG responses persist for over 3 months.

## INTRODUCTION

Severe acute respiratory syndrome coronavirus 2 (SARS-CoV-2), the causative agent of coronavirus disease 2019 (COVID-19), has spread rapidly around the world since first identified in Wuhan, China, in December 2019 (*1*). On March 11^th^, 2020 the World Health Organization (WHO) declared COVID-19 a pandemic, which surpassed 1 million reported global deaths on September 28th, 2020 (*2*).

Currently, our understanding of antibody responses following infection with SARS-CoV-2 is limited (*3*–*5*). Specifically, we lack detailed descriptions and precise estimates concerning the magnitude and duration of responses, cross-reactivity with other coronaviruses and viral respiratory pathogens, and correlates of protective immunity following infection. A detailed characterization of antibody responses is needed to determine whether antibody-based tests can augment viral detection-based assays in the diagnosis of active or recent infection and to inform the design and interpretation of seroepidemiologic studies.

In this study, we characterize the kinetics and antibody isotype profile to the receptor binding domain (RBD) of the spike (S) protein of SARS-CoV-2 in a longitudinal cohort of North American patients infected with SARS-CoV-2, most of whom were hospitalized for COVID-19, and in pre-pandemic controls. We also examined how well these responses correlated with neutralizing antibody activity directed at the S protein. Additionally, we evaluated the cross-reactivity of these responses with other coronavirus RBDs and characterize assay performance using dried blood spots as an alternative to serum or plasma.

## RESULTS

### Study cohorts

Using an in-house enzyme linked immunosorbent assay (ELISA), we measured anti-RBD antibody responses in two cohorts: 1) symptomatic patients who tested positive for SARS-CoV-2 by PCR (n = 343) and 2) healthy (n = 1,515) and febrile controls (n = 33) collected prior to the SARS-CoV-2 pandemic. The majority of SARS-CoV-2 positive cases were severe (93% hospitalized, 53% requiring ICU level care, 13% died), male (62%), and older (median age: 59) (Table 1, Figure S1). Most pre-pandemic controls were younger (median age: 37) and female (66%). Plasma and/or serum was collected at multiple time points for most patients (63%; n=216), with 34% (n=118) having ≥ 4 samples. Forty-two percent of cases had a sample collected between 0-7 days after onset of symptoms (n=143), 55% had a sample between 8-14 days (n=189), 48% had a sample between 15-28 days (n=165), 35% had a sample between 29-45 days (n=121), 22% had a sample between 46-60 days (n=76), and 10% had a sample > 60 days (n=35). The last sample was collected 122 days post-symptom onset. Twenty-six (8%) cases were immunosuppressed (e.g., on methotrexate, rituximab, etc.), and we did not expect them to mount a robust immune response.

**Table 1:** Individual characteristics of PCR-positive SARS-CoV-2 cases and pre-pandemic controls.

Characteristic	Pre-pandemic Controls* (N=1,548)	PCR-positive Cases (N=343)
Age		
Median [IQR]	37 [30–54]	59 [45–71]
<65 years (%)	1,386 (90)	213 (62)
65+ years (%)	162 (10)	130 (38)
Female (%)	1,024 (66)	132 (38)
Race or ethnic group^¥^		
White (%)	NA	125 (36)
Black or African American (%)	NA	34 (10)
Hispanic or Latino (%)	NA	121 (35)
Asian, American Indian, Alaska Native or Other (%)	NA	30 (9)
Immunosuppressed (%)	NA	26 (8)
Severity^†^		
Not Hospitalized (%)	NA	24 (7)
Hospitalized, no ICU (%)	NA	138 (40)
Hospitalized, required ICU (%)	NA	137 (40)
Died due to COVID-19 (%)	NA	43 (13)

*Pre-pandemic controls included healthy adults (n=274), patients undergoing routine serology testing (n=1241), and patients presenting with other known febrile illnesses (n = 33), including 13 with bacteremia (e.g., *S. aureus*, *S. pneumoniae*, *E. coli*, or *K. pneumoniae* confirmed by standard microbiologic techniques), 4 with babesiosis (confirmed by microscopy and/or PCR), 1 with presumed scrub typhus, and 15 with viral respiratory infections (e.g., influenza [7], parainfluenza [4], respiratory syncytial virus [3], and metapneumovirus [1] confirmed by PCR or direct fluorescent antibody test).

^¥^Data available for 310 cases.

^†^Data available for 342 cases.

### Kinetics of anti-SARS-CoV-2 RBD antibody responses

**Fig. 1:**
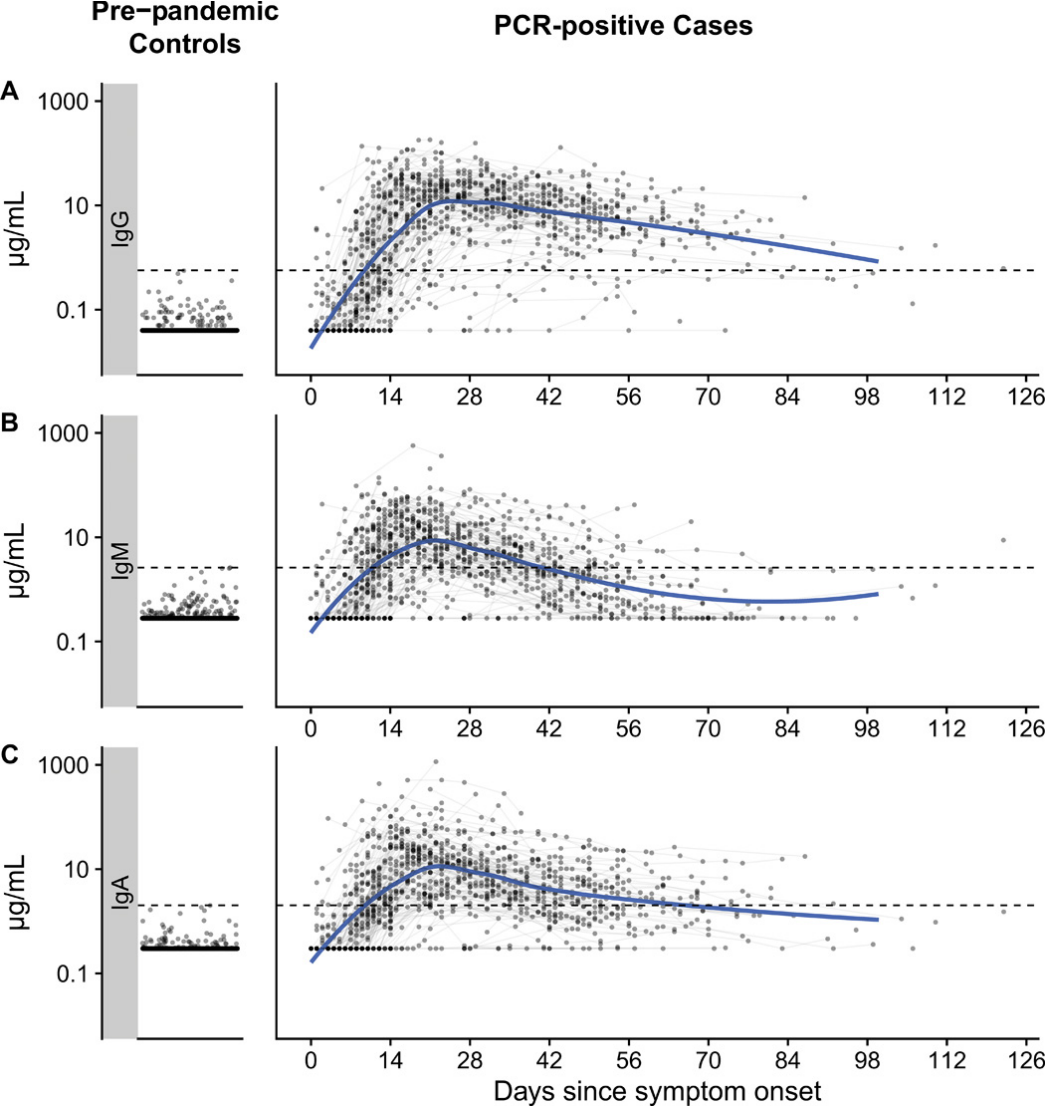
Measurement of IgG, IgM, IgA against SARS-CoV-2 spike protein receptor binding domain among pre-pandemic controls and PCR positive cases. Each dot represents a unique measurement of an isotype (Row A: IgG, Row B: IgM, Row C: IgA) in pre-pandemic controls (left panels) and PCR positive cases (right panels). The blue line is a loess smooth nonparametric function. Black dashed lines indicate the maximum concentration (μg/mL) found among pre-pandemic controls (IgG: 0.57, IgM: 2.63, IgA: 2.02). Horizontal jitter was introduced into the pre-pandemic controls. The limit of detection (μg/mL) was 0.04 for IgG, 0.28 for IgM, and 0.30 for IgA.

If followed for more than 14 days since symptom onset, most cases (92%) had at least one IgG measurement higher than seen among any pre-pandemic control (Fig. 1). From days 5 to 14, there was a sharp rise in RBD-specific antibodies of all isotypes, and IgG measurements continued to rise until day 25 after the onset of symptoms (Figure S2A). The population average IgA and IgM responses peaked less than a week earlier than IgG and then declined toward concentrations measured in pre-pandemic samples (Figure S2 and S3). IgG antibody responses also began to wane, but at a slower rate. Among 117 cases with ≥ 4 measurements, the individual peak IgM measurement often occurred before that of IgG (before: 55%, simultaneous: 38%) and simultaneously with that of IgA (before: 28%, simultaneous: 53%). Among hospitalized patients, the population average trajectory differed little between severity levels; the average IgG concentrations among hospitalized cases admitted to the ICU were higher than hospitalized cases not admitted to the ICU (Figure S2B). Concentrations of all isotypes were lower among immunosuppressed individuals (Figure S2C).

### Accuracy of RBD antibodies for identifying recent SARS-CoV-2 infection

Each antibody isotype was indicative of infection, and the area under the receiver operating curve (AUC) for each antibody isotype increased to above 98% during the period of 15-28 days after symptom onset (Table 2). The AUC remained high for IgG (99%) and IgA (98%) after 28 days but began to fall for IgM (93%). Using test cutoffs set to ensure no false positives within the pre-pandemic samples (i.e., 100% within sample specificity), we found that the sensitivity of IgG antibodies rose from 7% (≤7 days) to 95% after 14 days of symptoms. The sensitivity of IgA and IgM rose to 90% and 81% 2-4 weeks post-symptom onset but dropped after 4 weeks to 66% and 44%, respectively. Through ten-fold cross-validation, we found that the mean specificity for each isotype was 99.9% (fold-specific range: 99.4 - 100%).

**Table 2:** Predictive accuracy of individual isotypes for classifying controls and cases across time.

Isotype	Days since symptom onset	AUC (95% CI)	Sensitivity (95% CI)
IgG	≤7 days	0.68 (0.66–0.70)	0.07 (0.03–0.12)
	8-14 days	0.91 (0.89–0.92)	0.51 (0.43–0.58)
	15-28 days	0.99 (0.99–1.00)	0.95 (0.92–0.98)
	>28 days	0.99 (0.99–1.00)	0.95 (0.91–0.98)
IgA	≤7 days	0.63 (0.61–0.65)	0.07 (0.03–0.11)
	8-14 days	0.87 (0.85–0.89)	0.44 (0.38–0.51)
	15-28 days	0.98 (0.97–0.98)	0.89 (0.84–0.94)
	>28 days	0.98 (0.97–0.98)	0.60 (0.51–0.68)
IgM	≤7 days	0.60 (0.58–0.62)	0.08 (0.03–0.13)
	8-14 days	0.87 (0.85–0.89)	0.55 (0.48–0.62)
	15-28 days	0.98 (0.97–0.99)	0.86 (0.81–0.92)
	>28 days	0.93 (0.91–0.94)	0.51 (0.43–0.59)

The isotype cut-offs chosen for calculating sensitivity were the maximum value found among pre-pandemic controls (IgG: 0.57 μg/mL, IgM: 2.63 μg/mL, IgA: 2.02 μg/mL). Bootstrap 95% confidence intervals are shown in parentheses.

### Combining multiple isotype measurements to improve accuracy

We found the accuracy of serologic identification of recent infections could be slightly improved by adding measurements of IgM and/or IgA to IgG at the earlier phases of infection (Table S2; Figure S4). Using random forest models to combine measurements of different isotypes, we estimated a cvAUC of 92% for IgG & IgM and 91% for IgG & IgA at 8-14 days post-symptom onset. These models provide an estimate of the contribution of each antibody isotype, as well as an approximation of the maximum predictive value of combined measures of anti-RBD IgG, IgA and IgM responses. While all isotypes contributed nearly equally to identifying recent infection antibody profiles in the early phase of illness, IgG responses were the most indicative of infection 8 or more days after the onset of symptoms (Figure S5). Using the pre-determined thresholds for seropositivity for each antibody isotype, out of the 357 samples collected during early infection (< 14 days post symptom onset), we were able to correctly identify an additional 19 (5%) cases among the IgG negative samples by adding IgM, 21 (6%) by adding IgA, and 33 (9%) by adding both IgM and IgA. When accounting for class imbalance in the random forest procedure, similar results were obtained (Figure S6, Figure S7).

### Estimation of time to seroconversion and seroreversion for each isotype

**Fig. 2:**
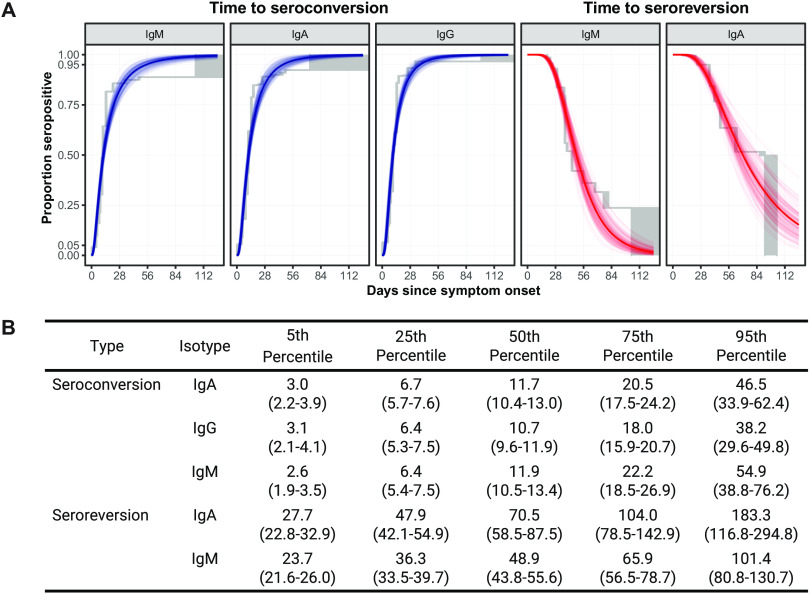
Parametric and nonparametric model estimates of time to seroconversion and seroreversion for each isotype. A) The isotype cut-offs chosen for seroconversion were the maximum concentration (μg/mL) found among pre-pandemic controls (IgG: 0.57, IgM: 2.63, IgA: 2.02). The solid line represents the estimated cumulative distribution function of the time to seroconversion or reversion with 100 bootstrapped fits shown as transparent lines. The parametric accelerated failure time models assume a log-normal time-to-event distribution. Nonparametric estimates shown in grey were calculated using the Turnbull method. Only 3 individuals seroreverted for IgG, so no model is included. B) The table indicates the estimated average number of days since onset of symptoms it takes for a percentage of cases to seroconvert or serorevert. Bootstrap 95% confidence intervals are shown in parentheses.

Using the cutoffs defined earlier, we estimated the distribution of the time required to become seropositive (seroconversion) and return to becoming seronegative (seroreversion). Overall, 324 (94%) individuals had more than 1 measurement for every 28 days of follow-up. Of the 159 cases with samples after 20 days post-symptoms, most had evidence of seroconversion for all isotypes (IgG: 96%, IgM: 88%, IgA: 89%). The estimated median time to seroconversion from symptom onset was comparable across antibody isotype: 10.7 days (95% CI: 9.6-11.9) for IgG, 11.7 days (10.4-13.0) for IgA and 11.9 (10.5-13.4 days) for IgM (Fig. 2). On average, we estimated the median time to seroconversion among hospitalized patients to be over four days earlier as compared to nonhospitalized patients for all isotypes; men and those aged <65 years also seroconverted more quickly on average (Table S3).

Of seroconverted cases with samples 46 days post-symptoms or after, most eventually had IgM (45/61) and IgA (30/64) seronegative measurements. The median time to seroreversion for IgM was 48.9 days (95% CI: 43.8 – 55.6), with the first 5% seroreverting by 23.7 days (95% CI: 21.6 – 26.0). We estimated a slightly later median seroreversion time for IgA of 70.5 days (95% CI: 58.5 - 87.5), with the first 5% seroreverting by 27.7 days (95% CI: 22.8–32.9, Fig. 2). Only 3 of 70 cases had evidence of seroreversion for IgG. All 3 patients who seroreverted for IgG required ICU level care, however 2 of the 3 did not have robust IgG responses (peak IgG measurement < 2 μg/mL, 1 of whom was immunosuppressed).

### Association between RBD responses and the development of neutralizing antibodies targeting the S protein

**Fig. 3:**
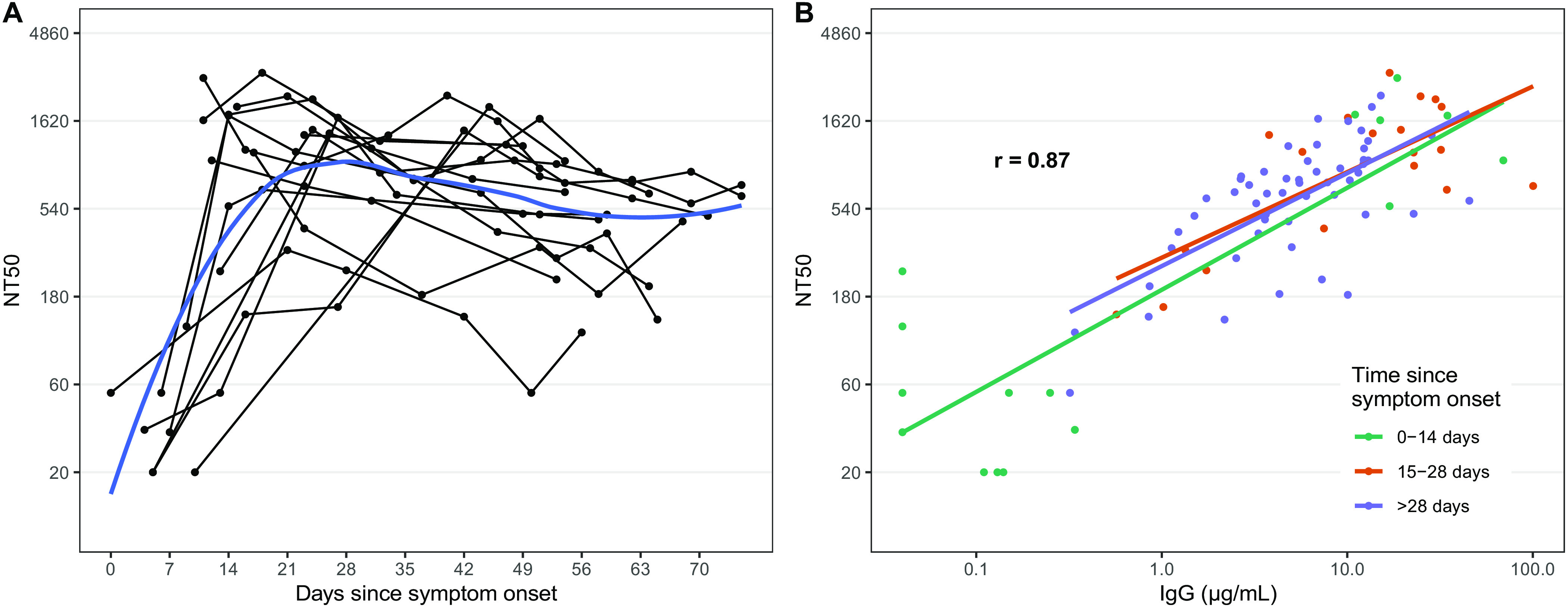
SARS-CoV-2 pseudovirus neutralization antibody titers in symptomatic PCR positive cases and correlation with anti-RBD IgG responses. A) Each point represents a measurement of 50% neutralizing titer (NT50). Lines connect measurements from the same individual and a loess smooth function is shown in blue. B) The overall repeated measures correlation coefficient (r) is shown. Lines represent simple linear models for each time period.

We measured pseudoneutralizing antibodies targeting the SARS-CoV-2 S protein in 88 samples from 15 individuals collected between 0 and 75 days post-symptoms (Fig. 3). Over the course of infection, all individuals tested developed detectable neutralizing antibodies (NAb). NAb titers were correlated with the concentration of anti-RBD IgG (r = 0.87). Of note, similar to anti-RBD IgG responses, NAb titers plateaued and remained detectable at later time points despite the more rapid decline of IgA and IgM responses.

### Evaluation of cross-reactivity with other coronaviruses

We evaluated antibody responses to RBDs derived from spike proteins of endemic human coronaviruses (CoVs) (i.e., HKU1, 229E, OC43, and NL63), severe acute respiratory syndrome coronavirus (SARS-CoV-1) and Middle East Respiratory Syndrome coronavirus (MERS-CoV) (Figure S8). Antibody responses to the endemic CoVs were comparable between pre-pandemic controls and individuals with COVID-19 at all phases of infection, demonstrating a lack of cross-reactivity. Although a few individuals with SARS-CoV-2 infection had increasing levels of antibodies to endemic CoVs over time, which could be explained by cross-reactive anamnestic responses/ immunologic memory, the majority stayed the same. Thus, overall, we did not observe a detectable cross-reactive response to the RBDs of the endemic human coronaviruses across the population of individuals infected with SARS-CoV-2. In contrast, we did observe significant cross-reactivity to SARS-CoV-1 RBD in individuals with COVID-19, but no significant cross-reactive responses to the MERS-CoV RBD. Of note, there were three pre-pandemic controls (samples collected prior to October 2019) with IgA cross-reactivity to SARS-CoV-1.

### Comparison of plasma responses to dried blood spots (DBS)

Since DBS could be used in large serosurveys where venous blood may be logistically challenging to collect and process, we also evaluated the assay with simulated dried blood spot eluates in a subset of patients (n= 20 at two timepoints; 40 samples) and pre-pandemic controls (n=20). The anti-RBD IgG DBS measurements had a high degree of linear correlation in both cases and control plasma (r = 0.99, Figure S9). While the classification of all samples was the same between DBS and plasma samples (100% classification concordance), values between the two sample types diverged more at low titer values.

## DISCUSSION

In this study, we found that antibodies against the RBD region of the S protein were accurate indicators of recent severe SARS-CoV-2 infection. The presence of IgG antibodies targeting SARS-CoV-2 RBD was a highly sensitive (95%) marker of infection after 14 days from onset of illness. This is consistent with a growing body of data which demonstrate that measurement of anti-RBD antibodies can accurately classify individuals recently infected with SARS-CoV-2 (*6*–*9*). Because this study was conducted in a large cohort of individuals with known SARS-CoV-2 infection (N=343) and controls (N=1548) it provides a robust measure of the accuracy of anti-RBD antibodies.

These findings also add to emerging evidence on the persistence and decay of antibody responses following SARS-CoV-2 infection. IgM and IgA responses to RBD were short-lived and most individuals seroreverted within two and a half months after the onset of illness. However, IgG antibodies persisted at detectable levels in patients beyond 90 days after symptom onset, and seroreversion was only observed in a small percentage of individuals. The concentration of these anti-RBD IgG antibodies was also highly correlated with pseudovirus NAb titers, which also demonstrated minimal decay. The observation that IgG and neutralizing antibody responses persist is encouraging, and suggests the development of robust systemic immune memory in individuals with severe infection. This is similar to a study that reported on anti-RBD antibodies in 121 North American convalescent plasma donors up to 82 days from symptom onset (*10*) and a study of 1,197 Icelanders who remained seropositive by 2 pan-IgG SARS-CoV-2 antibody assays 120 days after qPCR diagnosis of SARS-CoV-2 (*9*). However, these findings differ with other recent studies suggesting a more rapid waning in anti-RBD titers following mild or asymptomatic SARS-CoV-2 infection (*11*, *12*).

RT-PCR based detection of SARS-CoV-2 is sensitive early in the first week after the onset of symptoms (*13*), and our results suggest that the detection of antibodies against the SARS-CoV-2 RBD by ELISA, even when utilizing all isotypes, is not likely to contribute significantly to the early diagnosis of COVID-19. However, beyond two weeks after symptom onset, supplementing viral detection assays with antibody-based testing methods clearly increases sensitivity in diagnosing recent infection (*14*, *15*), particularly as the sensitivity of RT-PCR for SARS-CoV-2 infection wanes (*12*). In particular, our results demonstrate that the earlier seroreversion of IgA and IgM responses will be helpful in distinguishing older infections from recent ones. Thus, the measurement of multiple isotypes, taking into account the early decay of IgA and IgM, is likely to be critical in interpreting the results of serosurveys and epidemiologic studies to estimate the time from infection. All considered, these findings suggest clearly defined applications for serologic testing of RBD responses in both clinical and public health/surveillance settings.

Testing for anti-SARS-CoV-2 RBD antibodies can also be applied in seroepidemiologic studies, even in areas of low prevalence, given their excellent specificity and defined kinetics. Variation in the performance of commercial serologic tests and confusion about the role of antibodies as biomarkers of past infection versus protective immunity has led to widespread misperception that antibody testing may be inaccurate (*16*, *17*). In contrast, our study, based on a very large sample of cases and controls, should provide significant confidence in the contribution of serologic measures in public health efforts to improve epidemiological investigations (*18*) and to provide high-resolution estimates of infection incidence across geographies and populations. In addition, the lack of cross-reactivity of antibodies to SARS-CoV-2 RBD with common cold coronaviruses provides additional data supporting the specificity of the assay.

One limitation of our study was that our cohort of individuals with SARS-CoV-2 infection was skewed toward adults with severe disease or with risk factors for disease progression. It is important to study the kinetics and in particular the decay of antibody responses in individuals with severe infection for several reasons. First, the magnitude and duration of the responses in individuals with severe infection likely provide an estimate of the upper bounds of the achievable immune response and the development of B cell memory following natural infection. Second, these findings are expected to have significant implications for protective immunity in a population which clearly is vulnerable to poor outcomes when exposed. However, caution is required in generalizing these results to those with less severe infection. Individuals with mild or asymptomatic infection have been shown to develop less robust antibody responses (*12*), which may lead to false negatives if our proposed assay thresholds are used. Individuals with mild or asymptomatic infection may also serorevert more quickly than symptomatic individuals. The gradation of responses by disease severity has been found in other infections, including SARS-CoV-2 and MERS-CoV infection (*19*). An association between disease severity and the kinetics of the antibody response is also suggested by our finding that individuals with more severe disease, who required ICU-level care, seroconverted earlier than individuals who did not require ICU-level support.

While anti-RBD antibodies accurately identify individuals with recent SARS-CoV-2 infection, it remains unknown whether these responses are associated with protection against subsequent infection. In many human challenge studies of common cold coronavirus infection, the presence of neutralizing antibodies has been associated with protection against symptomatic infection and decreased viral shedding (*5*). In addition, in vaccinated rhesus macaques challenged with SARS-CoV-2 infection, neutralizing antibodies directed at the S protein were also a strong correlate of protective immunity (*20*). Thus, neutralization titers, in the absence of other known markers, have become a *de facto* immunologic marker of protection pending further investigation. In this context, it is notable that anti-RBD IgG antibodies were strongly correlated with the same neutralizing antibodies that were associated with protection in vaccinated macaques (*20*). This correlation with neutralizing titers was stronger than observed for other previously tested commercial serologic assays (*21*), and both anti-RBD and neutralizing antibodies persisted over a 2.5 month follow-up period.

Our results, therefore, provide strong support for the application of anti-RBD antibodies as a marker of recent SARS-CoV-2 infection as well as new and detailed information related to the specificity and decay kinetics of the anti-RBD responses. The testing approach used meets the CDC’s guidelines for serologic testing (*22*) and has the potential to facilitate accurate diagnosis in clinical settings and the implementation of population-based studies of previous infection globally. While the association between anti-RBD-IgG and neutralizing titers and the persistence of these antibodies at late time points is encouraging, further work is needed to define the optimal antibody-mediated correlates of protective immunity.

## MATERIALS AND METHODS

### Study design

We evaluated the magnitude and kinetics of the early human antibody response to the receptor binding domain of SARS-CoV-2 spike protein, with the additional objective of evaluating the specificity and sensitivity of these antibody responses for identifying individuals with recent infection. Thus, we measured antibody concentration in blood samples obtained from confirmed patients with symptomatic SARS-CoV-2 infection and from control individuals whose samples were collected prior to the pandemic. IgG, IgA and IgM antibody concentrations were measured by ELISA using recombinant SARS-CoV2 RBD in all samples. In a subset of samples, neutralizing antibody responses directed against the spike protein were also measured using a lentivirus pseudoneutralization model. From these data, we modeled the classification accuracy for each individual isotype and combinations of isotypes at different time points, and the temporal dynamics of seroconversion and seroreversion following the onset of symptoms.

### Sample collection

We obtained plasma and/or serum samples, collected for routine clinical care, from individuals with PCR-confirmed SARS-CoV-2 infection presenting, with fever and/or viral respiratory symptoms from March to April 2020 and who met criteria for RT-PCR testing. Testing criteria for SARS-CoV-2 changed over time, but primarily included patients with severe symptoms requiring hospital admission, although those who had other risk factors for disease progression (e.g., were age 60 or older, had diabetes, or were immunocompromised), or who worked or lived in a setting where infection control requirements dictated a need for testing. Additional serum/plasma samples collected September 2015 to December 2019 prior to the SARS-CoV-2 pandemic included healthy adults seen at the MGH Immunization and Travel Clinic prior to travel, patients undergoing routine serology, and patients presenting with other known febrile illnesses. Plasma samples, except for the routine serology samples, were heat-inactivated at 56°C for one hour prior to analysis.). Patient demographic information, lab results, and clinical outcomes were extracted from the electronic medical record. Patients were considered immunosuppressed if they had underlying immunosuppressive condition (e.g., HIV with CD4 count less than 200) or were on an immunosuppressive/immunomodulating agent at the time of their admission (e.g., methotrexate, rituximab) All research was approved by the Institutional Review Board for Human Subjects Research at MGH.

### Dried blood spots (DBS)

Seventy-two microliters of single donor, seronegative whole blood collected from sodium heparin tubes (Becton, Dickinson, NJ), was spiked with 8 μl heat-inactivated plasma (10% of the whole blood volume) to maintain the relative whole blood composition. Assuming plasma is 50% of the whole blood volume, the spiked plasma was 18.18% of the final plasma volume. Whole blood (40 μL) was spotted onto Whatman 903 Protein Saver cards (GE Healthcare, Cardiff, UK) in replicate and allowed to dry overnight at room temperature. Two 6-mm^2^ punches from the DBS card (5 μL plasma per punch) were placed in 133 μL PBS-0.05% Tween 20 pH 7.4 (Sigma-Aldrich, St. Louis, MO) and incubated overnight at 4°C with gentle agitation eluates were then recovered after centrifugation. The total dilution of the spiked plasma in DBS eluate was assumed to be 1:73.15, which accounts for the initial dilution from spiking (1:5.5) and the further dilution during DBS elution (1:13.3)

### Enzyme-linked immunosorbent assay (ELISA)

The ELISA assays measured IgG, IgA, and IgM responses to the receptor binding domain of the spike protein (RBD) from SARS-CoV-2 [GenBank: MN975262], Middle East Respiratory Syndrome (MERS) virus [GenBank: AFY13307.1], SARS-CoV-1 [GenBank: AAP13441.1], and common cold coronaviruses HKU1 [GenBank: AAT98580.1], OC229E [GenBank: AAK32191], OC43 [GenBank:AAT84362], and NL63 [GenBank: AKT07952]. RBD sequences were cloned into pVRC vector followed by expression in mammalian cells Expi293 cells (ThermoFisher Scientific, Waltham,MA) with a C-terminal streptavidin-binding peptide (SBP)-His8X tag, and purified over TALON resin (Takara, Mountain View, CA) followed by size exclusion chromatography and cleavage of the His tag. RBD-specific antibody concentrations (μg/mL) were quantified using isotype-specific anti-RBD monoclonal antibodies. The RBDs were expressed in Expi293F suspension cells with a C-terminal SBP-His8X tag, and purified using affinity chromatography and then size exclusion chromatography prior to removal of the His tag as described previously (*23*). Briefly, 384 well Nunc MaxiSorp plates (Invitrogen, Carlsbad, CA) were coated by adding 50 μL of RBD in carbonate buffer (1 μg/mL) and incubating for 1 hour at room temperature (RT). Plates were then blocked for 30 min at RT with 5% nonfat milk in tris-buffered saline (TBS). Diluted samples (1:100 in TBS with 5% milk, 0.5% Tween) were added to the plate (25 μL/well) and incubated for 1 hour at 37°C with shaking. Serial 4-fold dilutions to 1:6400 were also included for individuals with high titers. At the end of incubation, samples were washed 5 times with 1X high salt TBS. Subsequently, goat anti-human IgA, IgG, and IgM- horseradish peroxidase conjugated secondary antibodies diluted (Jackson ImmunoResearch) at 1:10000 (IgG, IgM) or 1:5000 (IgA) in 5% milk in TBST were added to plates (25 μL/well) and incubated at RT with shaking for 30 min followed by 5X 1X high salt TBS washes and a last wash with 1X TBS. Bound secondaries were detected using 1-step Ultra TMB (tetramethylbenzidine; ThermoScientific, Waltham, MA, 25 μL/well). Plates were incubated at RT for 5 min in the dark before addition of 2 N sulfuric acid stop solution (25 μL/well). The optical density (OD) was read at 450 nm and 570 nm on a plate reader. OD values were adjusted by subtracting the 570 nm OD from the 450 nm OD. We used a standard curve of the anti-SARS-CoV-2 monoclonal, CR3022 (*24*), to calculate the concentration of anti-RBD IgG, IgA, and IgM expressed in μg/mL. Note: For the DBS and plasma comparisons the starting concentration was 1:200.

### Pseudovirus neutralization assay

To determine the SARS-CoV-2 neutralization activity of our plasma samples, we used a lentivirus pseudoneutralization model as previously described (*20*), which is a strong correlate of protective immunity in challenged rhesus macaques (*25*). We expressed results from this assay as the antibody titer required to neutralize 50% of the SARS-CoV-2 pseudovirus (NT50).

### Statistical analysis

#### Single isotype thresholds

We first explored how cutoffs of individual isotypes (IgM, IgG and IgA) performed in identifying previously infected individuals. We compared measurements from pre-pandemic controls, with those taken at any time, ≤7 days, 8-14 days, 15-28 days, and >28 days after the onset of symptoms. We estimated the AUC for each isotype and time period combination and calculated bootstrap 95% confidence intervals. Using the isotype cutoffs defined by the maximum concentration (μg/mL) found among the full set of pre-pandemic controls (IgG: 0.57, IgM: 2.63, IgA: 2.02), we estimated sensitivity and bootstrap 95% confidence intervals. We also evaluated how setting a cutoff defined by maximum concentration would affect specificity through ten-fold cross-validation.

#### Random forest classification models

We explored how combining multiple isotype-specific responses with random forest classification models, which allows for complex nonlinear interactions between isotypes, performed identifying previously infected individuals. Using a previously described cross-validation procedure (*26*), we allocated both cases and controls into ten equally sized groups (i.e., folds) and calculated a pooled cross-validated AUC (cvAUC). We also assessed variable importance within these different models using a permutation test–based metric, mean decrease in accuracy. To investigate the impact of class imbalance (i.e., the fact that we had many more negative controls than positives) on our model performance metrics, we investigated the effect of downsampling controls to have the same number as cases on model performance.

#### Analysis of time to seroconversion and seroreversion

We limited our analysis to individuals who had at least one measurement for every 28 days of follow-up (i.e., between symptom onset and their last measurement). For individuals with fluctuations around the pre-defined cutoff (N=6), the time to the first event was used in the analysis. Using individual level interval-censored data, we fitted nonparametric (i.e Turnbull’s Estimator) and parametric accelerated failure time models using the icenReg R package (*27*). All time-to-event data were assumed to be log-normal distributed. Bootstrapped 95% confidence intervals were estimated by sampling individuals with replacement.

All analyses were completed using R (Version 3.6.1) within Rstudio (Version 1.2.5019).

## Supplementary Materials

immunology.sciencemag.org/cgi/content/full/5/52/eabe0367/DC1 Figure S1: Number of PCR positive cases with a sample taken during each week since symptom onset. Figure S2. Smooth average measurements of IgG, IgM, and IgA against SARS-CoV-2 spike protein receptor binding domain among PCR positive cases across time. Figure S3. Individual trajectories for 16 randomly selected individuals with 4 or more measurements. Figure S4. Measurements of IgG, IgM, and IgA against SARS-CoV-2 spike protein receptor binding domain among pre-pandemic controls and symptomatic PCR positive cases. Figure S5. Receiver operating characteristic curve from random forest models and isotype contributions. Figure S6. Confusion matrices and out-of-bag error estimates for random forest models. Figure S7. Confusion matrices and out-of-bag error estimates for random forest models with downsampled controls. Figure S8. Measurements of IgG, IgA, and IgM against the RBD of other coronaviruses among pre-pandemic controls and PCR positive cases. Figure S9. Correlation between plasma and dried blood spot measurements (DBS). Table S1. Full amino acid sequences of the coronavirus receptor-binding domains (RBDs) used in this study. Table S2. Predictive accuracy of multiple isotypes for classifying controls and cases over time since symptom onset. Table S3. Parametric estimates of median time to seroconversion for each isotype by different patient characteristics. Table S4. Raw data file (Excel spreadsheet).

## References

[1] M. Lipsitch, D. L. Swerdlow, L. Finelli, Defining the Epidemiology of Covid-19 - Studies Needed. N. Engl. J. Med. 382, 1194–1196 (2020). 10.1056/NEJMp2002125 32074416

[2] WHO Coronavirus Disease (COVID-19) Dashboard. https://covid19.who.int/. Accessed 28th September,2020.

[3] T. Zohar, G. Alter, Dissecting antibody-mediated protection against SARS-CoV-2. Nat. Rev. Immunol. 20, 392–394 (2020). 10.1038/s41577-020-0359-5 32514035 PMC7278217

[4] M. Döhla, C. Boesecke, B. Schulte, C. Diegmann, E. Sib, E. Richter, M. Eschbach-Bludau, S. Aldabbagh, B. Marx, A.-M. Eis-Hübinger, R. M. Schmithausen, H. Streeck, Rapid point-of-care testing for SARS-CoV-2 in a community screening setting shows low sensitivity. Public Health 182, 170–172 (2020). 10.1016/j.puhe.2020.04.009 32334183 PMC7165286

[5] A. T. Huang, B. Garcia-Carreras, M. D. T. Hitchings, B. Yang, L. C. Katzelnick, S. M. Rattigan, B. A. Borgert, C. A. Moreno, B. D. Solomon, L. Trimmer-Smith, V. Etienne, I. Rodriguez-Barraquer, J. Lessler, H. Salje, D. S. Burke, A. Wesolowski, D. A. T. Cummings, A systematic review of antibody mediated immunity to coronaviruses: Kinetics, correlates of protection, and association with severity. Nat. Commun. 11, 4704 (2020). 10.1038/s41467-020-18450-4 32943637 PMC7499300

[6] L. Premkumar, B. Segovia-Chumbez, R. Jadi, D. R. Martinez, R. Raut, A. Markmann, C. Cornaby, L. Bartelt, S. Weiss, Y. Park, C. E. Edwards, E. Weimer, E. M. Scherer, N. Rouphael, S. Edupuganti, D. Weiskopf, L. V. Tse, Y. J. Hou, D. Margolis, A. Sette, M. H. Collins, J. Schmitz, R. S. Baric, A. M. de Silva, The receptor binding domain of the viral spike protein is an immunodominant and highly specific target of antibodies in SARS-CoV-2 patients. Sci. Immunol. 5, eabc8413 (2020). 10.1126/sciimmunol.abc8413 32527802 PMC7292505

[7] L. Ren, G. Fan, W. Wu, L. Guo, Y. Wang, X. Li, C. Wang, X. Gu, C. Li, Y. Wang, G. Wang, F. Zhou, Z. Liu, Q. Ge, Y. Zhang, H. Li, L. Zhang, J. Xu, C. Wang, J. Wang, B. Cao, Antibody Responses and Clinical Outcomes in Adults Hospitalized with Severe COVID-19: A Post hoc Analysis of LOTUS China Trial. Clin. Infect. Dis. ciaa1247 (2020). 10.1093/cid/ciaa1247 32840287 PMC7499517

[8] K. M. McAndrews, D. P. Dowlatshahi, J. Dai, L. M. Becker, J. Hensel, L. M. Snowden, J. M. Leveille, M. R. Brunner, K. W. Holden, N. S. Hopkins, A. M. Harris, J. Kumpati, M. A. Whitt, J. J. Lee, L. L. Ostrosky-Zeichner, R. Papanna, V. S. LeBleu, J. P. Allison, R. Kalluri, Heterogeneous antibodies against SARS-CoV-2 spike receptor binding domain and nucleocapsid with implications for COVID-19 immunity. JCI Insight 5, 142386 (2020). 32796155 10.1172/jci.insight.142386 PMC7526535

[9] D. F. Gudbjartsson, G. L. Norddahl, P. Melsted, K. Gunnarsdottir, H. Holm, E. Eythorsson, A. O. Arnthorsson, D. Helgason, K. Bjarnadottir, R. F. Ingvarsson, B. Thorsteinsdottir, S. Kristjansdottir, K. Birgisdottir, A. M. Kristinsdottir, M. I. Sigurdsson, G. A. Arnadottir, E. V. Ivarsdottir, M. Andresdottir, F. Jonsson, A. B. Agustsdottir, J. Berglund, B. Eiriksdottir, R. Fridriksdottir, E. E. Gardarsdottir, M. Gottfredsson, O. S. Gretarsdottir, S. Gudmundsdottir, K. R. Gudmundsson, T. R. Gunnarsdottir, A. Gylfason, A. Helgason, B. O. Jensson, A. Jonasdottir, H. Jonsson, T. Kristjansson, K. G. Kristinsson, D. N. Magnusdottir, O. T. Magnusson, L. B. Olafsdottir, S. Rognvaldsson, L. le Roux, G. Sigmundsdottir, A. Sigurdsson, G. Sveinbjornsson, K. E. Sveinsdottir, M. Sveinsdottir, E. A. Thorarensen, B. Thorbjornsson, M. Thordardottir, J. Saemundsdottir, S. H. Kristjansson, K. S. Josefsdottir, G. Masson, G. Georgsson, M. Kristjansson, A. Moller, R. Palsson, T. Gudnason, U. Thorsteinsdottir, I. Jonsdottir, P. Sulem, K. Stefansson, Humoral Immune Response to SARS-CoV-2 in Iceland. N. Engl. J. Med. •••, (2020). 10.1056/NEJMoa2026116 32871063 PMC7494247

[10] F. A. Ania Wajnberg, Adolfo Firpo, Deena Altman, Mark Bailey, Mayce Mansour, Meagan McMahon, Philip Meade, Damodara Rao Mendu, Kimberly Muellers, Daniel Stadlbauer, Kimberly Stone, Shirin Strohmeier, Judith Aberg, David Reich, Florian Krammer, Carlos Cordon-Cardo, SARS-CoV-2 infection induces robust, neutralizing antibody responses that are stable for at least three months. medRxiv (2020), https://www.medrxiv.org/content/10.1101/2020.07.14.20151126v1

[11] F. Javier Ibarrondo, A. Jennifer, Fulcher, David Goodman-Meza, Julie Elliott, Christian Hofmann, Mary A. Hausner, Kathie G. Ferbas, Nicole H. Tobin, Grace M. Aldrovandi, Otto O. Yang, Rapid Decay of Anti-SARS-CoV-2 Antibodies in Persons with Mild Covid-19. N. Engl. J. Med. (2020). 10.1056/NEJMc2025179 PMC7397184 32706954

[12] Q.-X. Long, X.-J. Tang, Q.-L. Shi, Q. Li, H.-J. Deng, J. Yuan, J.-L. Hu, W. Xu, Y. Zhang, F. J. Lv, K. Su, F. Zhang, J. Gong, B. Wu, X.-M. Liu, J.-J. Li, J.-F. Qiu, J. Chen, A.-L. Huang, Clinical and immunological assessment of asymptomatic SARS-CoV-2 infections. Nat. Med. 26, 1200–1204 (2020). 10.1038/s41591-020-0965-6 32555424

[13] L. M. Kucirka, S. A. Lauer, O. Laeyendecker, D. Boon, J. Lessler, Variation in False-Negative Rate of Reverse Transcriptase Polymerase Chain Reaction-Based SARS-CoV-2 Tests by Time Since Exposure. Ann. Intern. Med. 173, 262–267 (2020). 10.7326/M20-1495 32422057 PMC7240870

[14] L. Guo, L. Ren, S. Yang, M. Xiao, D. Chang, F. Yang, C. S. Dela Cruz, Y. Wang, C. Wu, Y. Xiao, L. Zhang, L. Han, S. Dang, Y. Xu, Q.-W. Yang, S.-Y. Xu, H.-D. Zhu, Y.-C. Xu, Q. Jin, L. Sharma, L. Wang, J. Wang, Profiling Early Humoral Response to Diagnose Novel Coronavirus Disease (COVID-19). Clin. Infect. Dis. 71, 778–785 (2020). 10.1093/cid/ciaa310 32198501 PMC7184472

[15] S. E. F. Yong, D. E. Anderson, W. E. Wei, J. Pang, W. N. Chia, C. W. Tan, Y. L. Teoh, P. Rajendram, M. P. H. S. Toh, C. Poh, V. T. J. Koh, J. Lum, N. M. Suhaimi, P. Y. Chia, M. I. Chen, S. Vasoo, B. Ong, Y. S. Leo, L. Wang, V. J. M. Lee, Connecting clusters of COVID-19: An epidemiological and serological investigation. Lancet Infect. Dis. 20, 809–815 (2020). 32330439 10.1016/S1473-3099(20)30273-5 PMC7173813

[16] Antibody Test, Seen as Key to Reopening Country, Does Not Yet Deliver in The New York Times, (Published April 19, 2020).

[17] Antibody tests for Covid-19 wrong up to half the time, CDC in *CNN*. (May 26, 2020).

[18] Q. B. Zhen Zhang M, Shisong Fang, Lan Wei, Xin Wang, Jianfan He, Yongsheng Wu BS1, Xiaojian Liu MMed,Wei Gao MMed,Renli Zhang, Wenfeng Gong, Qiru Su,Andrew S Azman,Justin Lessler, Xuan Zou. (2020). Insights into the practical effectiveness of RT-PCR testing for SARS-CoV-2 from serologic data, a cohort study (2020). Published online September 08, 2020. https://doi.org/10.1101/2020.09.01.20182469 PMC7816573 33495759

[19] Ko. Jae-Hoon, A. Marcel, Müller, Hyeri Seok, Ga Eun Park, Ji Yeon Lee, Sun Young Cho, Young Eun H, Jin Yang Baek, So Hyun Kim, Ji-Man Kang, Yae-Jean Kim, Ik Joon Jo, Chi Ryang Chung, Myong-Joon Hahn, Christian Drosten, Cheol-In Kang,a Doo Ryeon Chung, Jae-Hoon Song, Eun-Suk Kang, and Kyong Ran Peck, Serologic responses of 42 MERS-coronavirus-infected patients according to the disease severity. Diagn. Microbiol. Infect. Dis. 89, 106–111 (2017). 28821364 10.1016/j.diagmicrobio.2017.07.006 PMC7127792

[20] A. Chandrashekar, J. Liu, A. J. Martinot, K. McMahan, N. B. Mercado, L. Peter, L. H. Tostanoski, J. Yu, Z. Maliga, M. Nekorchuk, K. Busman-Sahay, M. Terry, L. M. Wrijil, S. Ducat, D. R. Martinez, C. Atyeo, S. Fischinger, J. S. Burke, M. D. Slein, L. Pessaint, A. Van Ry, J. Greenhouse, T. Taylor, K. Blade, A. Cook, B. Finneyfrock, R. Brown, E. Teow, J. Velasco, R. Zahn, F. Wegmann, P. Abbink, E. A. Bondzie, G. Dagotto, M. S. Gebre, X. He, C. Jacob-Dolan, N. Kordana, Z. Li, M. A. Lifton, S. H. Mahrokhian, L. F. Maxfield, R. Nityanandam, J. P. Nkolola, A. G. Schmidt, A. D. Miller, R. S. Baric, G. Alter, P. K. Sorger, J. D. Estes, H. Andersen, M. G. Lewis, D. H. Barouch, SARS-CoV-2 infection protects against rechallenge in rhesus macaques. Science 369, 812–817 (2020). 10.1126/science.abc4776 32434946 PMC7243369

[21] R. A. D. Elena Criscuolo, Marta Strollo, Serena Rolla, Alessandro Ambrosi, Massimo Locatelli, Roberto Burioni, Nicasio Mancini, Massimo Clementi, Nicola Clementi, Poor correlation between antibody titers and neutralizing activity in sera from SARS-CoV-2 infected subjects. medRxiv (2020), https://www.medrxiv.org/content/10.1101/2020.07.10.20150375v1 10.1002/jmv.26605 PMC7675753 33064340

[22] Centers for Disease Control and Prevention, Interim Guidelines for COVID-19 Antibody Testing at https://www.cdc.gov/coronavirus/2019-ncov/lab/resources/antibody-tests-guidelines.html. (2020). (2020). Accessed 27th, July 2020. (2020).

[23] M. Norman, T. Gilboa, A. F. Ogata, A. M. Maley, L. Cohen, Y. Cai, J. Zhang, J. E. Feldman, B. M. Hauser, T. M. Caradonna, B. Chen, A. G. Schmidt, G. Alter, R. C. Charles, E. T. Ryan, D. R. Walt, Ultra-Sensitive High-Resolution Profiling of Anti-SARS-CoV-2 Antibodies for Detecting Early Seroconversion in COVID-19 Patients. *medRxiv*, (2020), https://www.medrxiv.org/content/10.1101/2020.04.28.20083691v1, Published online, May 02,2020.

[24] C. Wang, W. Li, D. Drabek, N. M. A. Okba, R. van Haperen, A. D. M. E. Osterhaus, F. J. M. van Kuppeveld, B. L. Haagmans, F. Grosveld, B.-J. Bosch, Publisher Correction: A human monoclonal antibody blocking SARS-CoV-2 infection. Nat. Commun. 11, 2511 (2020). 10.1038/s41467-020-16256-y 32366817 PMC7198537

[25] J. Yu, L. H. Tostanoski, L. Peter, N. B. Mercado, K. McMahan, S. H. Mahrokhian, J. P. Nkolola, J. Liu, Z. Li, A. Chandrashekar, D. R. Martinez, C. Loos, C. Atyeo, S. Fischinger, J. S. Burke, M. D. Slein, Y. Chen, A. Zuiani, F. J. N. Lelis, M. Travers, S. Habibi, L. Pessaint, A. Van Ry, K. Blade, R. Brown, A. Cook, B. Finneyfrock, A. Dodson, E. Teow, J. Velasco, R. Zahn, F. Wegmann, E. A. Bondzie, G. Dagotto, M. S. Gebre, X. He, C. Jacob-Dolan, M. Kirilova, N. Kordana, Z. Lin, L. F. Maxfield, F. Nampanya, R. Nityanandam, J. D. Ventura, H. Wan, Y. Cai, B. Chen, A. G. Schmidt, D. R. Wesemann, R. S. Baric, G. Alter, H. Andersen, M. G. Lewis, D. H. Barouch, DNA vaccine protection against SARS-CoV-2 in rhesus macaques. Science 369, 806–811 (2020). 10.1126/science.abc6284 32434945 PMC7243363

[26] J. E. Bryant, A. S. Azman, M. J. Ferrari, B. F. Arnold, M. F. Boni, Y. Boum, K. Hayford, F. J. Luquero, M. J. Mina, I. Rodriguez-Barraquer, J. T. Wu, D. Wade, G. Vernet, D. T. Leung, Serology for SARS-CoV-2: Apprehensions, opportunities, and the path forward. Sci. Immunol. 5, eabc6347 (2020). 10.1126/sciimmunol.abc6347 32430309

[27] C. Anderson-Bergman, icenReg: Regression Models for Interval Censored Data in R. (2017). https://www.jstatsoft.org/article/view/v081i12

